# Long non-coding RNA CASC9 promotes gefitinib resistance in NSCLC by epigenetic repression of DUSP1

**DOI:** 10.1038/s41419-020-03047-y

**Published:** 2020-10-14

**Authors:** Zhenyao Chen, Qinnan Chen, Zhixiang Cheng, Jingyao Gu, Wenyan Feng, Tianyao Lei, Jiali Huang, Jiaze Pu, Xin Chen, Zhaoxia Wang

**Affiliations:** 1 Cancer Medical Center, The Second Affiliated Hospital of Nanjing Medical University, Nanjing, Jiangsu People’s Republic of China; 2 Department of Oncology, The Fourth Affiliated Hospital of Nanjing Medical University, Nanjing, Jiangsu People’s Republic of China

**Keywords:** Non-small-cell lung cancer, Oncogenesis

## Abstract

Resistance to epidermal growth factor receptor tyrosine kinase inhibitors (EGFR-TKIs), such as gefitinib, has greatly affected clinical outcomes in non-small cell lung cancer (NSCLC) patients. The long noncoding RNAs (lncRNAs) are known to regulate tumorigenesis and cancer progression, but their contributions to NSCLC gefitinib resistance remain poorly understood. In this study, by analyzing the differentially expressed lncRNAs in gefitinib-resistant cells and gefitinib-sensitive cells in the National Institute of Health GEO dataset, we found that lncRNA CASC9 expression was upregulated, and this was also verified in resistant tissues. Gain and loss of function studies showed that CASC9 inhibition restored gefitinib sensitivity both in vitro and in vivo, whereas CASC9 overexpression promoted gefitinib resistance. Mechanistically, CASC9 repressed the tumor suppressor DUSP1 by recruiting histone methyltransferase EZH2, thereby increasing the resistance to gefitinib. Furthermore, ectopic expression of DUSP1 increased gefitinib sensitivity by inactivating the ERK pathway. Our results highlight the essential role of CASC9 in gefitinib resistance, suggesting that the CASC9/EZH2/DUSP1 axis might be a novel target for overcoming EGFR-TKI resistance in NSCLC.

## Introduction

Non-small cell lung cancer (NSCLC) is one of the most common malignant tumors worldwide. Despite continuous developments and progress in therapeutic measures, such as surgery, radiotherapy, and drug therapy (including chemotherapy, targeted therapy, and immunotherapy), the prognosis of patients with advanced NSCLC is still poor^1,2^. The first generation of epidermal growth factor receptor tyrosine kinase inhibitors (EGFR-TKI), represented by gefitinib, could significantly prolong the median survival of patients with advanced lung cancer with EGFR-sensitive mutations and greatly improve their quality of life^3^. However, acquired resistance is inevitable with gefitinib, which leads to treatment failure^4^. Therefore, in-depth study of the mechanism of drug resistance to EGFR-TKIs, the search for genes related to drug resistance, and the exploration of ways to reverse drug resistance have recently become important topics in lung cancer treatment research.

As one type of non-coding RNA, long-chain non-coding RNA (lncRNA) can regulate gene expression on multiple levels, such as the epigenetic, transcription, and post-transcription levels^5–7^. For example, one study showed that LINC01234 activated VAV3 at the post-transcription level and repressed BTG2 at the transcription level, which subsequently led to epithelial–to–mesenchymal transition in NSCLC cells. Additionally, lncRNA SNHG20 promoted proliferation and migration by epigenetically silencing P21^8^.

lncRNAs also form complex regulatory networks in cells^9^, which provides a new research direction for drug resistance in NSCLC. lncRNAs play a key role in the development and formation of tumor resistance^10–13^. lncRNA UCA1 promotes gefitinib resistance by sponging miR-143 in NSCLC^14^, and lncRNA CCAT1 acts as an miR-218 sponge to increase gefitinib resistance by targeting HOXA1^15^. Our previous study revealed that LINC01116 facilitates gefitinib resistance by downregulating IFI44^13^. As a result, lncRNAs have been highlighted as novel players in tumor resistance. However, only a small fraction of lncRNAs and their role in drug resistance have been elucidated, and much information remains unknown.

In the present study, we performed lncRNA profiling of GEO datasets and identified CASC9, which was overexpressed in gefitinib-resistant NSCLC cells and tissues. CASC9 promoted gefitinib resistance both in vitro and in vivo. We further uncovered the underlying mechanism and proposed that CASC9 may be a novel predictive biomarker and therapeutic target for NSCLC gefitinib resistance.

## Results

### CASC9 is overexpressed in gefitinib-resistant PC9/GR cells and correlated with acquired resistance to gefitinib

**Fig. 1:**
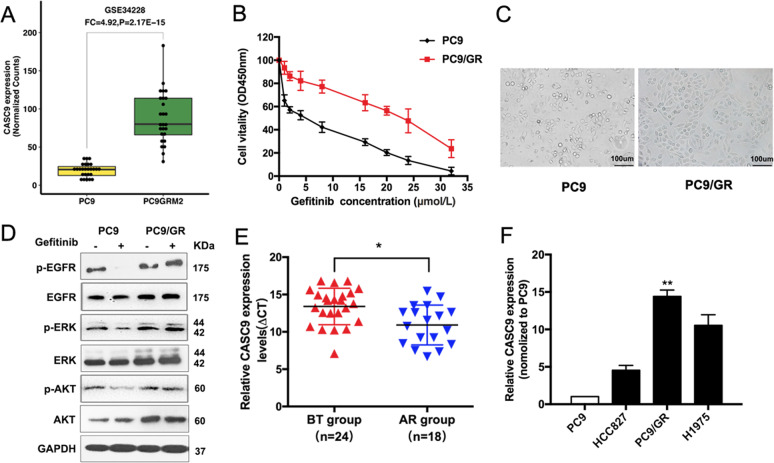
Relative CASC9 expression in gefitinib-resistant tissues and cell lines. **a** Data mining of altered CASC9 expression in microarray gene profiles (GSE34228). **b** IC50 values of gefitinib in PC9/GR and their respective parental PC9 cells was examined by CCK8 assay. **c** Observation the morphological differences of PC9/GR and PC9 cells by microscope. **d** The expression of p-EGFR, p-AKT, p-ERK, total EGFR, AKT, ERK, and GAPDH of PC9 and PC9/GR cells treated with 5 μM gefitinib were measured by western blot. **e** CASC9 was detected in BT group and AR group by qRT-PCR. The levels of CASC9 in AR tissues are significantly higher than that in BT tissues. The ΔCt value was determined by subtracting the GAPDH Ct value from the CASC9 Ct value. A smaller ΔCt value indicates higher expression. **f** CASC9 expression of PC9, HCC827, H1975 and PC9/GR cells was evaluated by qRT-PCR. *P < 0.05, **P < 0.01.

To identify the mechanisms that are involved in acquired resistance to EGFR-TKIs, we conducted an integrative analysis of lncRNAs/mRNA for PC9 and PC9/GR cells of GSE34228 from the GEO datasets. CASC9 expression was higher in PC9/GR cells with acquired resistance to gefitinib (Fig. 1a). To explore the correlation between CASC9 and EGFR-TKIs, we established gefitinib-resistant (GR) NSCLC cells by exposing PC9 cells to increasing concentrations of gefitinib for over 6 months. The inhibitory concentration of gefitinib yielding 50% cell viability (IC50) was calculated from linear dose–response curves using the CCK8 assay (Fig. 1b). We used high power microscopy to observe the morphology of PC9 and PC9/GR cells (Fig. 1c). Next, we evaluated the effects of gefitinib on the phosphorylation of EGFR, ERK, and PI3K/AKT, three major downstream pathways that activate in PC9/GR cells. Western blot results showed that after gefitinib treatment, there was no significant change in the phosphorylation of EGFR, ERK, and AKT in PC9/GR cells, while the phosphorylation of EGFR, ERK, and AKT in PC9 cells was significantly inhibited (Fig. 1d and Supplementary Fig. S1a). These findings suggested that there is a bypass in PC9/GR cells to activate the EGFR downstream signal. Furthermore, we examined CASC9 expression in developing resistance to EGFR-TKIs in advanced NSCLC patients, which were divided into two groups (Supplementary Table S1): before EGFR-TKI treatment (defined as the BT group) and acquired resistance to EGFR-TKIs (defined as the AR group). qRT-PCR analysis showed that CASC9 expression in the AR group was significantly higher than in the BT group (Fig. 1e). In NSCLC cell lines, CASC9 expression was higher in TKI acquired resistance cells (PC9/GR and H1975) than in the TKI-sensitive cell line (PC9 and HCC827) (Fig. 1f).

### CASC9 resensitizes gefitinib-resistant NSCLC to gefitinib in vitro

**Fig. 2:**
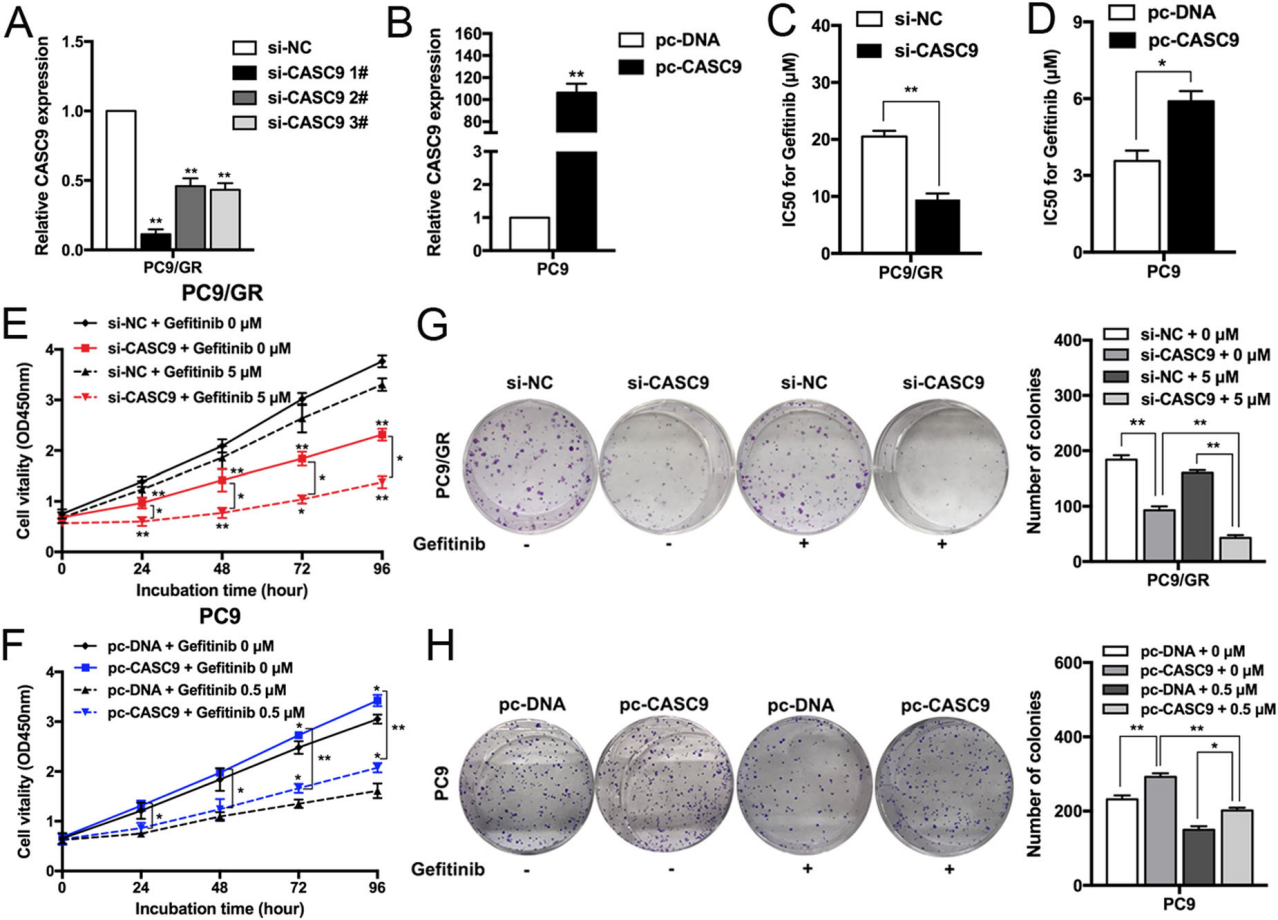
Downregulation/Upregulation of CASC9 increases/reduces the sensitivity of PC9/GR and PC9 cells to gefitinib. **a**, **b** qRT-PCR analysis of CASC9 expression in PC9 and PC9/GR cells overexpressing or depleted of CASC9. **c**, **d** CCK8 assays were measure the IC50 ability of CASC9-depleted or -overexpressing PC9/GR and PC9 after various concentration of gefitinib treatment for 72 h. **e**, **f** CCK8 assays were performed to determine the proliferation of CASC9-depleted or -overexpressing PC9/GR and PC9 cells treated with gefitinib. **g**, **h** Colony formation assays were used to evaluate the colony formation capacity of CASC9-depleted or -overexpressing PC9/GR and PC9 cells treated with gefitinib. *P < 0.05, **P < 0.01.

To explore the biological functions of CASC9 in the sensitivity of NSCLC cells, we transfected PC9/GR cells with CASC9-specific small interfering RNA (siRNA) to diminish its expression (Fig. 2a), and a CASC9 overexpression vector was transfected into parental PC9 cells to upregulate its expression (Fig. 2b). CCK8 assays showed that CASC9 downregulation significantly inhibited the IC50 value of PC9/GR cells to gefitinib (Fig. 2c), and that CASC9 overexpression induced PC9 cell resistance to gefitinib (Fig. 2d). To further confirm the role of CASC9 in gefitinib resistance, we constructed CASC9 overexpression vector with binding sites of si-CASC9 mutated. Co-transfection with CASC9 mutated vector and si-CASC9 showed higher IC50 value compared with co-transfection with empty vector and si-CASC9 (Supplementary Fig. S1b and c). Next, we determined the effects of CASC9 on cell proliferation. As shown in Fig. 2e, f, CCK8 proliferation assays showed that CASC9 downregulation significantly inhibited PC9/GR cell proliferation compared with the control cells and, conversely, CASC9 overexpression promoted PC9 cell proliferation with or without gefitinib treatment. Similar results were obtained when PC9/GR or PC9 cell clonogenic survival potential was measured with colony-forming assays (Fig. 2g, h).

**Fig. 3:**
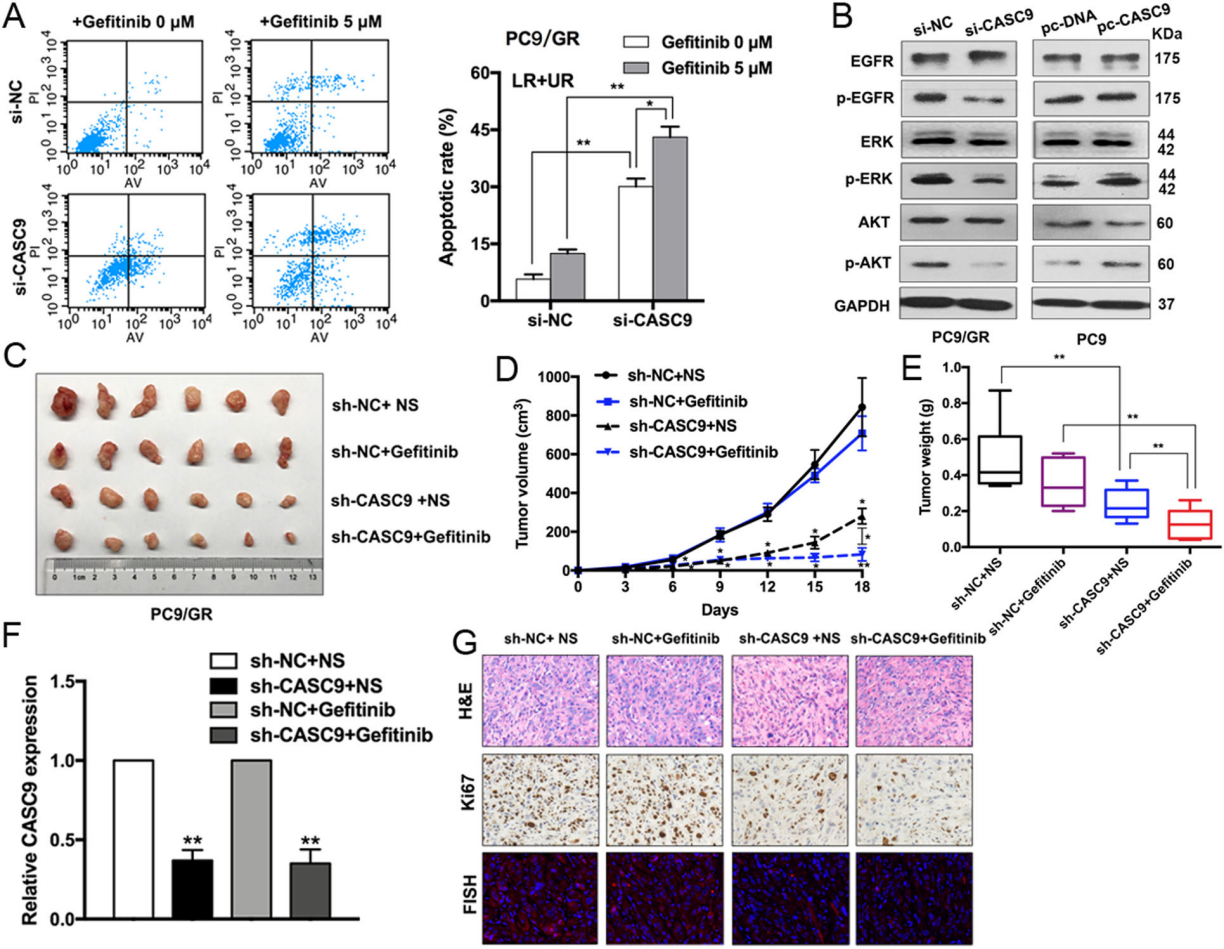
Downregulation of CASC9 reduces acquired gefitinib resistance in vivo. **a** FACS analysis of the effect of CASC9 down-regulation on PC9/GR cells apoptosis treated with gefitinib. **b** The expression of p-EGFR, p-AKT, p-ERK, total EGFR, AKT, ERK, and GAPDH of CASC9-depleted or -overexpressing PC9/GR and PC9 cells for 48 h were examined by western blot. **c** PC9/GR/sh-CASC9 or PC9/GR/Empty vector cells were injected into nude mice (*n* = 6). 9 days later, the mice were treated with normal saline or gefitinib (25.0 mg/kg) by the method of gavage. **d** Tumor volume vs time growth curves were measured every 3 days. **e** Tumor weight was measured after at 18 days after inoculation. **f** qRT-PCR analysis of relative expression of CASC9 in xenograft tumors. **g** Representative tumor sections derived from cells expressing PC9/GR/sh-CASC9 or PC9/GR/Empty vector treated with normal saline or gefitinib were subjected to H&E, Ki67 staining and FISH. *P < 0.05, **P < 0.01.

We also performed flow cytometry to investigate whether CASC9 is involved in regulating cell apoptosis. Compared with the control cells, CASC9 knockdown significantly increased the PC9/GR cell apoptotic rate with or without gefitinib treatment (Fig. 3a). We next examined whether CASC9 regulates ERK and AKT downstream signaling. CASC9 depletion resulted in significantly and markedly decreased phosphorylation levels of EGFR, AKT, and ERK in PC9/GR cells, whereas CASC9 overexpression dramatically increased EGFR, ERK, and AKT signaling in PC9 cells (Fig. 3b). Overall, these data confirmed that CASC9 can make drug-resistant cells sensitive to gefitinib in vitro.

### CASC9 knockdown suppresses gefitinib-resistant cancer development in vivo

To further validate the role of CASC9 in PC9/GR cell resistance to gefitinib in vivo, we injected the CASC9 stable knockdown PC9/GR cells or control cells into nude mice to employ a mouse xenograft model. As shown in Fig. 3c–e, CASC9 knockdown repressed tumor growth and weight, especially in cells treated with gefitinib. qRT-PCR analysis confirmed that CASC9 expression was inhibited in the excised tumors (Fig. 3f). Compared with the control tumors, sh-CASC9-derived tumors expressed lower levels of the proliferation marker Ki-67 as assessed by IHC staining and lower levels of CASC9 by fluorescence in situ hybridization (FISH) (Fig. 3g).

### CASC9 drives gefitinib resistance via increasing EZH2 and activating the ERK pathway

**Fig. 4:**
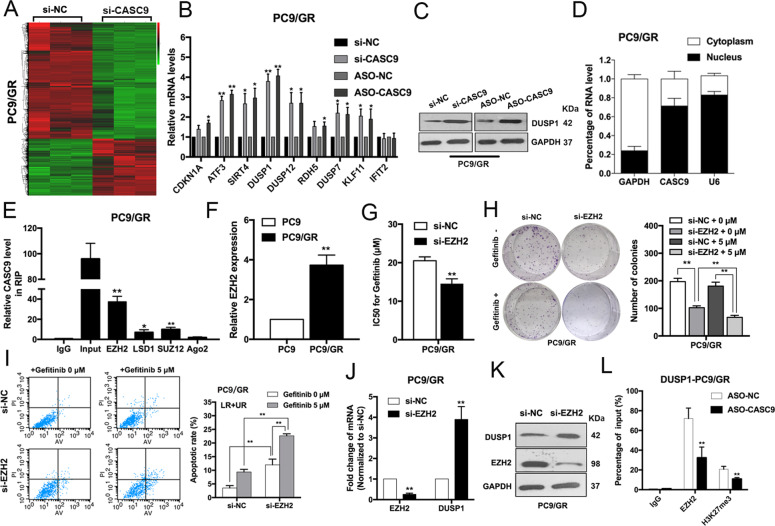
CASC9 represses DUSP1 expression by binding to EZH2. **a** Hierarchically clustered heatmap of differentially expressed genes in PC9/GR cells after transfection of CASC9 or control siRNAs. **b** Nine representative genes mRNA levels in PC9/GR cells depleted of CASC9. **c** Western blot analysis of DUSP1 expression in PC9/GR cells transfected with CASC9 siRNA and CASC9-ASO. **d** qRT-PCR analysis was performed to determine the subcellular localization of CASC9 in PC9/GR cells. **e** RIP assays were performed in PC9/GR cells to show CASC9 co-immunoprecipitation with EZH2, LSD1, SUZ12 and Ago2. **f** qRT-PCR analysis of EZH2 expression in PC9 and PC9/GR cells. **g** IC50 values of gefitinib in EZH2-depleted PC9/GR cells was examined by CCK8 assay. **h** Colony formation assays were used to evaluate the colony formation capacity of PC9/GR cells depleted of EZH2 treated with gefitinib. **i** FACS analysis of the effect of EZH2 down-regulation on PC9/GR cells apoptosis treated with gefitinib. **j**, **k** qRT-PCR and western blot analysis of EZH2 and DUSP1 mRNA and proteins expression in PC9/GR cells transfected with siRNA-NC or si-EZH2. **l** ChIP-qPCR assay showing EZH2 occupancy on the DUSP1 promoters was reduced by CASC9 knockdown. *P < 0.05, **P < 0.01.

The ERK pathway is of great importance in NSCLC cell proliferation, metastasis, and drug resistance^16^. To better understand the underlying molecular mechanism of CASC9 in acquired resistance to gefitinib, we used an RNA-sequencing analysis. To confirm the function of CASC9, we also designed LNA-ASO targeting CASC9. qRT-PCR studies confirmed that transfection with CASC9-ASO efficiently reduced CASC9 expression in PC9/GR cells (Supplementary Fig. S1d). As shown in Fig. 4a, expression of a large number of genes was upregulated in cells with CASC9 knockdown. We chose those genes involved in drug resistance to validate RNA-seq results. DUSP1 attracted our attention because of the highest-fold upregulation in CASC9 silencing PC9/GR cells (Fig. 4b). Western blot analysis subsequently confirmed that CASC9 knockdown significantly upregulated DUSP1 protein levels in PC9/GR cells (Fig. 4c). Additionally, we analyzed the distribution of CASC9 in PC9/GR cells using subcellular fractionation analyses. qRT-PCR analysis showed that CASC9 is distributed in both the cytoplasm and nucleus, but the ratio of CASC9 in the nucleus is higher (Fig. 4d), indicating that CASC9 may function as a regulator at the transcription level.

Recent studies have indicated that lncRNAs can regulate the expression of downstream targets by interacting with RNA binding proteins, such as EZH2, SUZ12, and LSD1^12,17^. To determine whether CASC9 regulates the potential targets by binding these proteins, we performed RNA immunoprecipitation (RIP) assays and confirmed that CASC9 is able to bind with EZH2, SUZ12, and LSD1. However, CASC9’s interaction with EZH2 was stronger, indicating that CASC9 interacted specifically with EZH2 in PC9/GR cells (Fig. 4e). To further study the interaction between CASC9 and EZH2, we examined the EZH2 expression by qRT-PCR and western blot. No significant changes were observed in si-CASC9 cells compared with negative control (Supplementary Fig. S1e, f).

EZH2, a core subunit of polycomb repressive complex 2 (PRC2), plays a vital role in treatment resistance in NSCLC^18,19^. To determine whether EZH2 is associated with gefitinib resistance, we examined EZH2 expression in PC9 and PC9/GR cells. The expression level of EZH2 was found to have a higher expression level in resistant cells of GSE34228 from GEO datasets (Supplementary Fig. S2a). Expression level of EZH2 was upregulated in PC9/GR cells compared with PC9 cells (Fig. 4f, Supplementary Fig. S2c, d). EZH2 knockdown restored gefitinib sensitivity, inhibited cell proliferation, and induced apoptosis in PC9/GR cells (Fig. 4g–i). In addition, we observed that the loss of EZH2 was related to DUSP1 upregulation at the mRNA and protein level (Fig. 4j, k). We then conducted chromatin immunoprecipitation assays and found that EZH2 could bind directly to the promoter regions of DUSP1 and induce H3K27 trimethylation. Furthermore, CASC9 knockdown decreased the binding of EZH2 and H3K27 trimethylation levels across the promoters of DUSP1 (Fig. 4l). These results suggest that CASC9 affects gefitinib resistance, at least partly, through the epigenetic repression of DUSP1 by interacting with EZH2 in PC9/GR cells.

### DUSP1 upregulation restored PC9/GR cell sensitivity to gefitinib

**Fig. 5:**
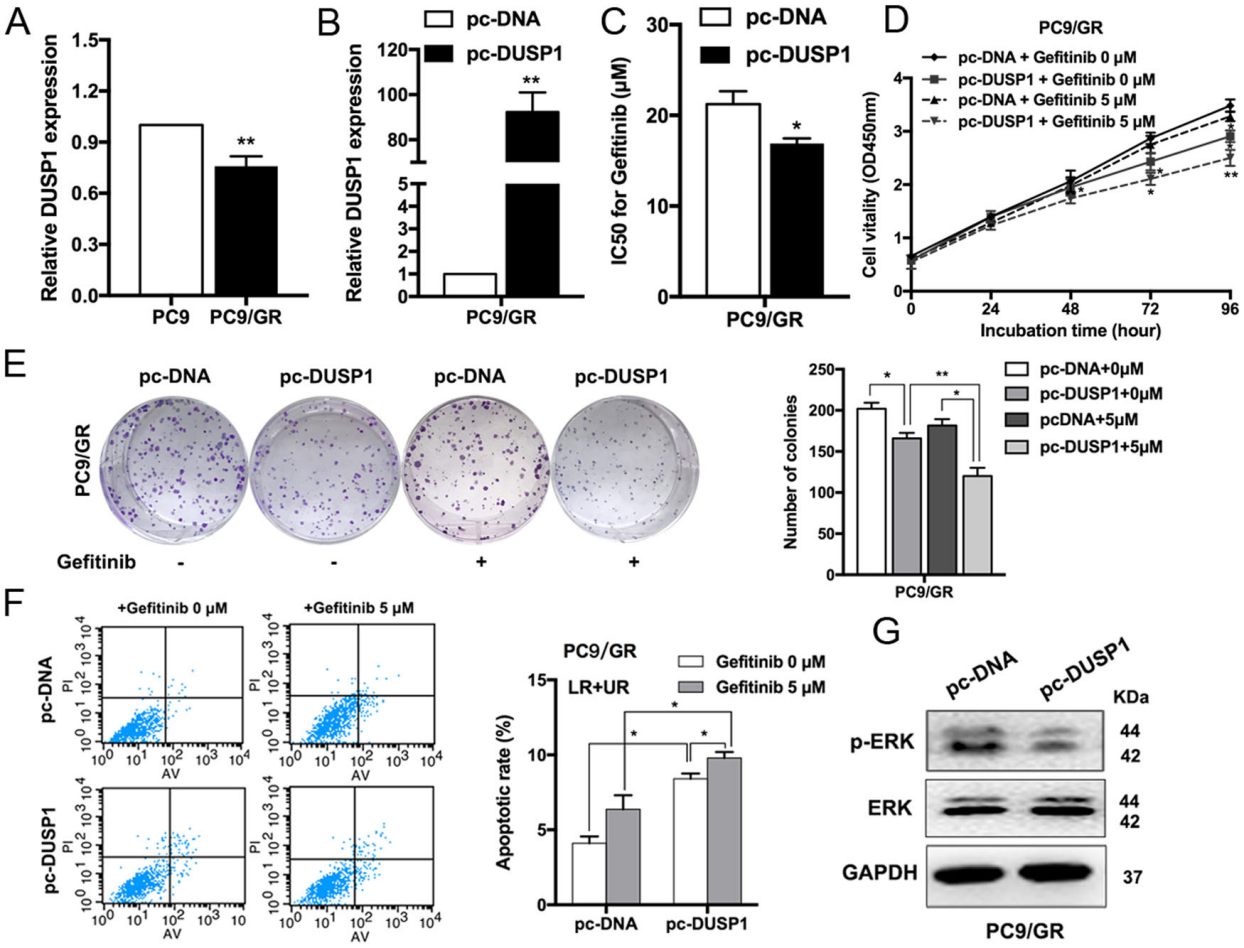
Upregulation of DUSP1 increases the sensitivity of PC9/GR cells to gefitinib and regulates ERK pathway. **a** qRT-PCR analysis of DUSP1 expression in PC9 and PC9/GR cells. **b** qRT-PCR analysis of DUSP1 mRNA levels in PC9/GR cells overexpressing DUSP1. **c** CCK8 assays were performed to determine the IC50 ability of DUSP1-overexpressing PC9/GR cells. **d**, **e** CCK8 and colony formation assays were performed to determine the proliferation of DUSP1-overexpressing PC9/GR cells treated with gefitinib. **f** FACS analysis of PC9/GR cells apoptosis overexpressing DUSP1 treated with gefitinib. **g** Western blot analysis of ERK and p-ERK proteins expression in PC9/GR cells transfected with DUSP1 vector. *P < 0.05, **P < 0.01.

To further verify the potential role of DUSP1 in PC9/GR cells, we examined its expression in PC9 and PC9/GR cells. DUSP1 was also found to be downregulated in resistant cells of GSE34228 from GEO datasets (Supplementary Fig. S2b). Expression level of DUSP1 was upregulated in PC9 cells compared with PC9/GR cells (Fig. 5a, Supplementary Fig. S2c, d). DUSP1 expression was upregulated when we used an overexpression vector (Fig. 5b), and DUSP1 overexpression reduced the IC50 of PC9/GR cells treated with gefitinib (Fig. 5c). CCK8 and colony formation assays revealed that DUSP1 overexpression inhibited PC9/GR cell proliferation with or without gefitinib treatment (Fig. 5d, e). Moreover, flow cytometry showed that the proportion of apoptotic cells increased significantly following treatment with a DUSP1 vector (Fig. 5f). We also discovered that DUSP1 overexpression markedly decreased the expression of phosphorylation-ERK (p-ERK), indicating that the ERK signaling pathway was inactivated, by western blot (Fig. 5g). Additionally, we detected the mRNA and protein levels of EZH2 and DUSP1 in the xenograft tumors after sh-CASC9 and gefitinib treatment. There were no significant differences of expression levels of EZH2 in those groups. DUSP1 was upregulated with knockdown of CASC9 in the presence or absence of gefitinib (Supplementary Fig. S2e–g).

### CASC9 contributes to acquired resistance to gefitinib and is partly dependent on regulating DUSP1

**Fig. 6:**
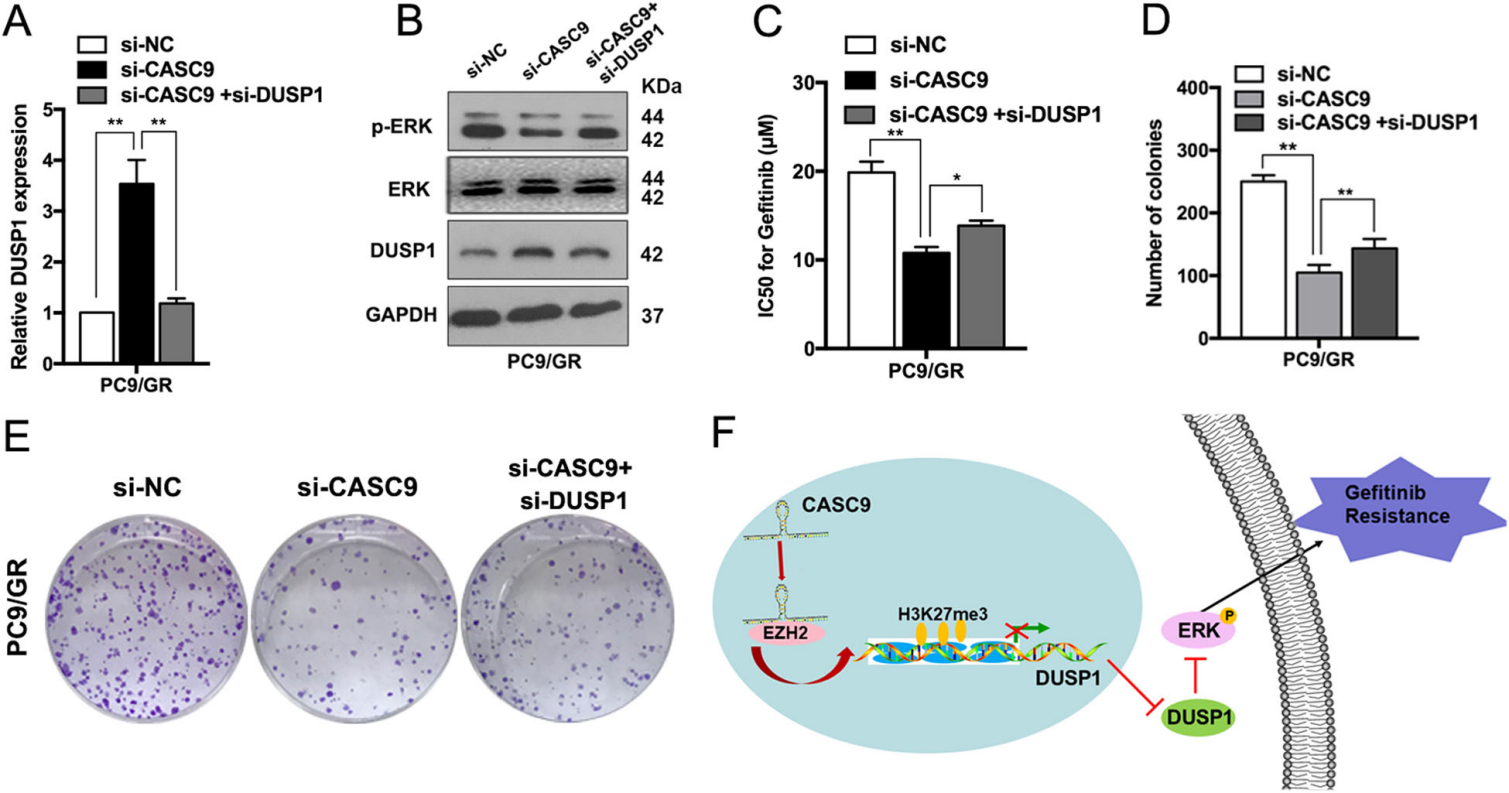
Downregulation of DUSP1 could rescue the effect of si-CASC9 on the sensitive of PC9/GR cells to gefitinib and regulate ERK signaling pathway. **a** qRT-PCR analysis detection of DUSP1 mRNA expression after co-transfection of PC9/GR cells with si-DUSP1 and si-CASC9. **b** ERK, p-ERK and DUSP1 protein levels were detected after co-transfection of PC9/GR cells with si-DUSP1 and si-CASC9. **c** CCK8 assays were performed to determine the IC50 ability of PC9/GR cells after co-transfection with si-DUSP1 and si-CASC9. **d**, **e** Colony formation assays were performed to determine the proliferation of PC9/GR cells after co-transfection with si-DUSP1 and si-CASC9. **f** Proposed model which medicated by CASC9 in gefitinib resistance of NSCLC. *P < 0.05, **P < 0.01.

Next, we identified whether suppressing DUSP1 expression could restore the effects of CASC9 knockdown on regaining gefitinib sensitivity in PC9/GR cells. As shown in Fig. 6a, si-CASC9 expression significantly increased DUSP1 mRNA levels, and these effects were reversed by co-expression of a DUSP1 inhibiter. The increased DUSP1 expression and decreased p-ERK protein induced by si-CASC9 were also partly rescued by co-transfection with a DUSP1 inhibitor (Fig. 6b). Moreover, si-DUSP1 reversed the CASC9 knockdown-induced decrease in the IC50 of gefitinib in PC9/GR cells (Fig. 6c). Furthermore, DUSP1 downregulation reversed the effects of si-CASC9 on proliferation capacity in PC9/GR cells (Fig. 6d, e). Collectively, these data indicated that the CASC9-EZH2-DUSP1 axis regulates p-ERK expression to promote gefitinib resistance in NSCLC (Fig. 6f).

## Discussion

The mechanism of EGFR-TKI acquired resistance is very complex. Currently, the reported reasons for EGFR-TKI acquired resistance are EGFR 20 exon T790M mutation, MET amplification, and phenotypic transformation of tumor cells^20–23^. However, secondary drug resistance in tumors is caused by the regulation of a multi-factor network, and there is still a lack of clinically applicable efficacy prediction markers and effective intervention strategies based on drug resistance molecular mechanisms^24^. Recent studies have found that ncRNA affects gene expression and translation at transcriptional and post-transcriptional levels and plays an important role in the development of drug resistance in tumor cells. In the present study, lncRNA CASC9 was screened and identified using public lncRNA expression profiles from the GEO datasets (GSE34228). We established a gefitinib-resistant cell line named PC9/GR, which has been described and reported previously^13^, to investigate the molecular mechanism of lncRNA-mediated gefitinib resistance. Consistent with results shown in the GEO datasets, CASC9 was expressed at a higher level in gefitinib-resistant cells and tissues compared with gefitinib-sensitive cells and tissues. In vitro and in vivo studies uncovered CASC9’s important role in gefitinib resistance. Although CASC9 was upregulated and played an oncogenic role in different types of cancers^25–28^, the function and mechanisms of CASC9 in NSCLC gefitinib resistance has never been investigated.

A large number of recent studies have shown that lncRNAs promote gefitinib resistance through a variety of mechanisms. For example, lncRNAs induced gefitinib resistance by functioning as a competing endogenous RNA^29^. By recruiting histone modification enzymes or interacting with transcription factors, lncRNAs can activate or suppress gene transcription and regulate the development of gefitinib resistance^30–32^. In this study, we found that CASC9 was able to bind to the histone modification enzyme, EZH2, to repress DUSP1 expression. EZH2 is the core subunit of the PRC2 complex, which inhibits target gene transcription via trimethylating of H3K27^12^. EZH2 is involved in treatment resistance in multiple cancers. For example, EZH2 epigenetically regulates the FADD/PARP1 axis, leading to TMZ resistance in glioma^33^; EZH2 activates cellular survival pathways, resulting in resistance to cisplatin^34^; and H3K27 mediated by EZH2 induces multidrug resistance in small cell lung cancer^35^. In the present study, EZH2 knockdown partially reversed gefitinib resistance, inhibited cell proliferation, and induced apoptosis in PC9/GR cells. Our results are in accordance with previous studies illustrating that EZH2 regulates gefitinib resistance in cancer cells^36^.

We also performed transcriptome sequencing to identify the target genes of CASC9, and we identified DUSP1 as a candidate. DUSP1 is reduced in various cancers, including liver cancer, pancreatic cancer, and lung cancer and this gene is considered a tumor suppressor that belongs to the MKP phosphatase family. DUSP1 plays an important role in regulating cell proliferation, tumorigenesis, and drug resistance^37–39^. Our findings indicated that EZH2 was able to bind directly to the promoter regions of DUSP1 and induce H3K27 trimethylation. CASC9 silenced DUSP1 transcription by recruiting EZH2 to the promoter region of DUSP1. DUSP1 overexpression reduced the level of p-ERK.

ERK signaling plays a critical role in TKI resistance in NSCLC^40^. The ERK-DUSP1 pathway is a phosphorylation cascade, in which ERK activation requires dual phosphorylation by a specific MAPK kinase. Each activated MAPK specifically targets many proteins, such as downstream kinases and transcription factors. Importantly, some studies have shown an association between ERK/DUSP1 pathway activation and invasive tumor phenotypes, characterized by inducing drug resistance and poor survival. In the current study, we also found that DUSP1 restored PC9/GR cell sensitivity to gefitinib by reducing activity in the ERK signaling pathway. Rescue experiments showed that DUSP1 knockdown reversed the effects of si-CASC9 on proliferation, gefitinib resistance, and ERK signaling activity. These results confirmed that the oncogenic function of CASC9 is partly dependent on repression of DUSP1 transcription.

In summary, the present study revealed that CASC9 promoted gefitinib resistance by recruiting the histone modification enzyme, EZH2, to repress DUSP1 expression epigenetically, which is involved in the ERK signaling pathway. These findings enhance our understanding of the CASC9/EZH2/DUSP1 axis in NSCLC gefitinib resistance.

## Materials and methods

### Specimen and cell Lines

A cohort of 42 NSCLC specimen tissues were collected by CT-guided percutaneous lung biopsy or fiberoptic bronchoscopy lung biopsy from advanced NSCLC patients who had either an exon 19 deletion (19DEL) or an exon 21-point mutation (L858R) in their EGFRs, and none of these patients received radiotherapy or chemotherapy before surgery. Among these 42 collected tissue samples, 24 were collected from patients before EGFR-TKIs treatment (defined as BT group), other 18 developed acquired resistance to EGFR- TKIs (defined as AR group). This study was approved by the Research Ethics Committee of The Second Affiliated Hospital of Nanjing Medical University and informed consent was obtained from all subjects. These specimens were immediately snap-frozen in liquid nitrogen and stored at −80 ^◦^C until required. The clinical information of the patients is summarized in Supplementary Table S1.

The NSCLC cell lines PC9, HCC827 and H1975 were purchased from the Institute of Biochemistry and Cell Biology at the Chinese Academy of Sciences (Shanghai, China). The gefitinib-resistant PC9 cell line (PC9/GR) was established by stepwise escalation method: parental PC9 cell was cultured with stepwise escalation of concentration of gefitinib from 5 nM to 5 μM over 6 months. PC9 and PC9/GR cells were cultured in DMEM, and HCC827 and H1975 were cultured in RPMI 1640 medium supplemented with 10% fetal bovine serum (FBS) and antibiotics (100 U/ml penicillin and 100 mg/ml streptomycin) in humidified with 5% CO_2_ at 37^◦^C. All cells were authenticated by STR profiling and tested for mycoplasma contamination.

### RNA extraction and qRT-PCR analyses

Total RNA was extracted from NSCLC tissues or cells using TRIzol reagent (Invitrogen, Carlsbad, CA) following the manufacturer’s protocol. The extracted RNA (1.0 μg) was reverse transcribed to cDNA by using a Reverse Transcription Kit (Takara, Dalian, China). Real-time PCR analyses were conducted using SYBR Green (Takara, Dalian, China). Glyceraldehyde-3-phosphate dehydrogenase (GAPDH) and U6 snRNA were used as endogenous controls. The relative fold change in expression was analyzed and expressed by the 2^−ΔΔCt^ method. The rest of primers were listed in Supplementary Table S2.

### Plasmid constructs and cell transfection

CASC9 and DUSP1 cDNA were synthesized according to their coding sequences and cloned into the expression vector pcDNA3.1. Plasmids were purified using DNA Midiprep Kits (Qiagen, Valencia, CA) and transfected into PC9 and PC9/GR cells using X-treme GENE HP DNA transfection reagent (Roche, Basel, Switzerland). Three CASC9-targeting small interfering RNAs (siRNAs) (Genepharma, Shanghai, China) and three CASC9-targeting antisense oligonucleotides (ASOs) (Qiagen, Valencia, CA) were transfected into PC9/GR cells using lipofectamine 3000 (Invitrogen, Carlsbad, CA) following the manufacturer’s manual. The sequences for the siRNAs and ASOs are listed in Supplementary Table S2. Cells were collected 48 h after transfection for quantitative real-time PCR and other individual experiments.

### Cell proliferation assays

Cell proliferation was measured using the Cell Counting Kit-8 (CCK8) (APExBIO, Houston, TX). PC9 and PC9/GR cells transfected with si-CASC9, si-NC, pc-DNA or pc-CASC9 were plated in 96-well microtiter plates at 1.0 × 10^3^/well, and incubated overnight. Then, the cells were treated with different concentrations of gefitinib (AstraZeneca, London, UK) for 72 h. Subsequently, 10 μl of CCK-8 was added to each well and incubated for 2 h, cellular viability was determined by measuring the absorbance of the converted dye at 450 nm. For the colony-formation assay, cells were harvested at 24 h after transfection, then seeded into 6-well plates maintaining in media containing 10% FBS and exposed to gefitinib for 48 h. Then, the drugs were washed away and the medium was replaced every 4 days. the colonies were fixed with methanol and stained with a 0.1% crystal violet (Sigma-Aldrich) for 15 min. Colonies were calculated. Each experiment was performed in triplicate.

### In vivo tumor formation assay

For in vivo tumor formation assay, stable CASC9-knockdown PC9/GR cells (5 × 10^6^) and control cells (5 × 10^6^) were inoculated into twenty-four 5-week-old male nude mice. 9 days after the tumor cell inoculation, mice bearing tumors were randomly assigned to 4 groups (each group with 6 mice) and gefitinib treatment was administered by oral gavage every day at a dose of 25 mg/kg. The tumor volumes and weights were measured every 3 days in mice. Tumor volume was calculated using the formula: *V* = 0.5 × length × width^2^. At 18 days post-injection, mice were euthanized, and the subcutaneous growth of each tumor was examined. All animal experiments were conducted with the approval of the Nanjing Medical University Institutional Committee for Animal Research and in conformity with national guidelines for the care and use of laboratory animals.

### Transcriptome sequencing

Total RNA from PC9/GR cells with CASC9 knockdown and control cells were isolated and quantified. We used a NanoDrop 2000 (Thermo Scientific, USA) to measure the concentration of each sample and assess the quality by Agilent2200 (Agilent, USA). Ion Proton Total RNA-Seq Kit v2 was used to establish the sequencing library of each RNA sample according to the protocol provided by the manufacturer (Life Technologies, USA). Data are available in Supplementary Table S3.

### Flow-cytometric assay

Cells were plated in 6-well plates, treated with gefitinib with a dose of 0 or 5 μM. A flow cytometry assay was performed as described previously^10^. The percentage of apoptotic cells was determined with Annexin V-PI/FITC staining. Cell-cycle progression was determined using propidium iodide staining.

### Chromatin immunoprecipitation (ChIP) assay

ChIP assay was performed according to manufacturer’s instruction (Millipore, USA) as described previously^8^. The antibodies for EZH2 and H3 trimethyl Lys 27 were obtained from Millipore.

### RNA immunoprecipitation (RIP) assay

RIP assay was performed according to manufacturer’s instruction (Millipore, USA) as described previously^12^. Antibodies for RIP assays of EZH2, LSD1, SUZ12 and Ago2 were purchased from Millipore.

### Western blot assay and antibodies

Cell lysates were electrophoretically separated by 10% SDS-PAGE, transferred to 0.22-mm PVDF membranes (Millipore, USA), and incubated with specific antibodies. Autoradiograms were quantified by densitometry (Tannon, China). GAPDH antibody was used as a control. Other antibodies were listed in Supplementary Table S2.

### Statistical analysis

Statistical analyses were performed using SPSS 20.0 (IBM, Armonk, NY, USA) and Prism software (GraphPad, La Jolla, CA, USA). LncRNA expression levels in tissues and cell lines were compared using the Mann–Whitney *U* test. For the remaining assays, differences between groups were assessed by paired, two-tailed Student’s *t*-test, Wilcoxon’s test, or *χ*^2^ test, as appropriate. *P* < 0.05 was considered statistically significant.

## Supplementary information

Supplementary Figure S1

Supplementary Figure S2

Supplementary Figure legends

Supplementary Table S1

Supplementary Table S2

Supplementary Table S3
