# Supplementary figures and images for: Long non-coding RNA CASC9 promotes gefitinib resistance in NSCLC by epigenetic repression of DUSP1
Source: Cell Death Dis. 2020 Oct 14;11(10):858. doi: 10.1038/s41419-020-03047-y (PMC7560854; doi:10.1038/s41419-020-03047-y)

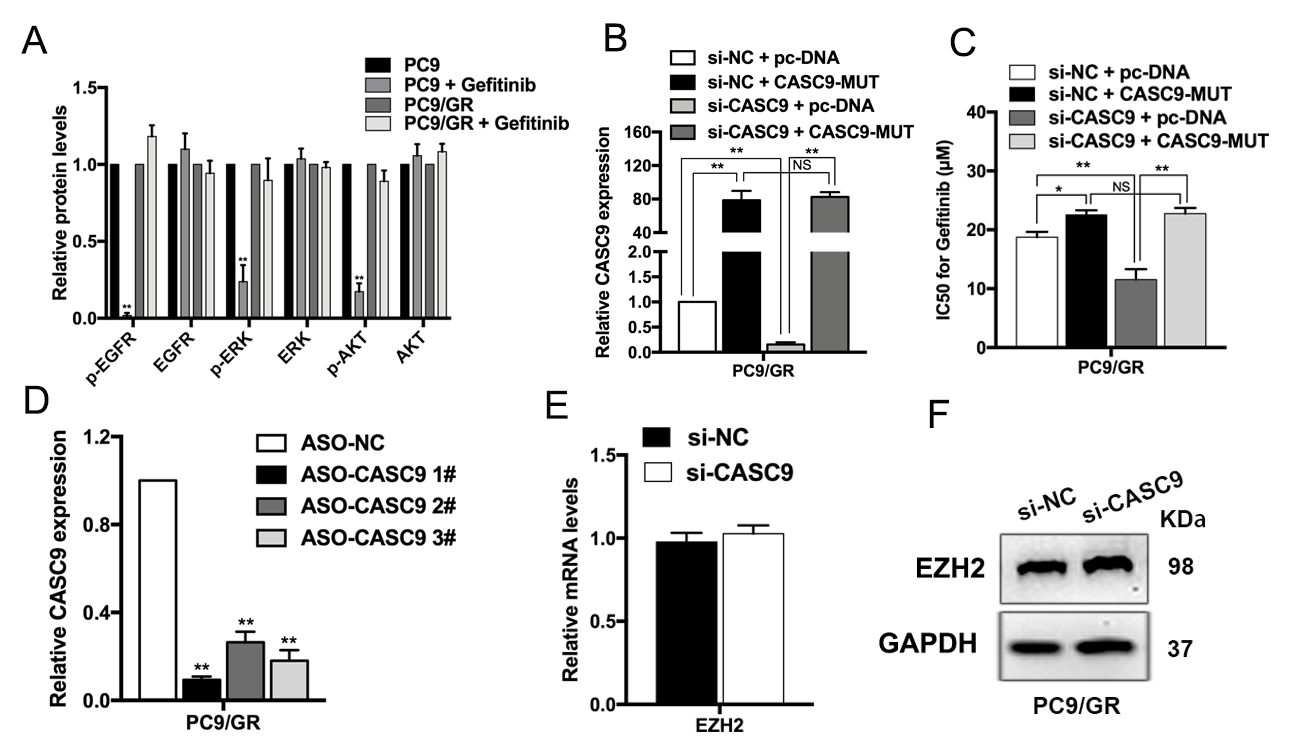

Supplement: Supplementary file 1 — Supplementary Figure S1 [file 41419_2020_3047_MOESM1_ESM.tif]

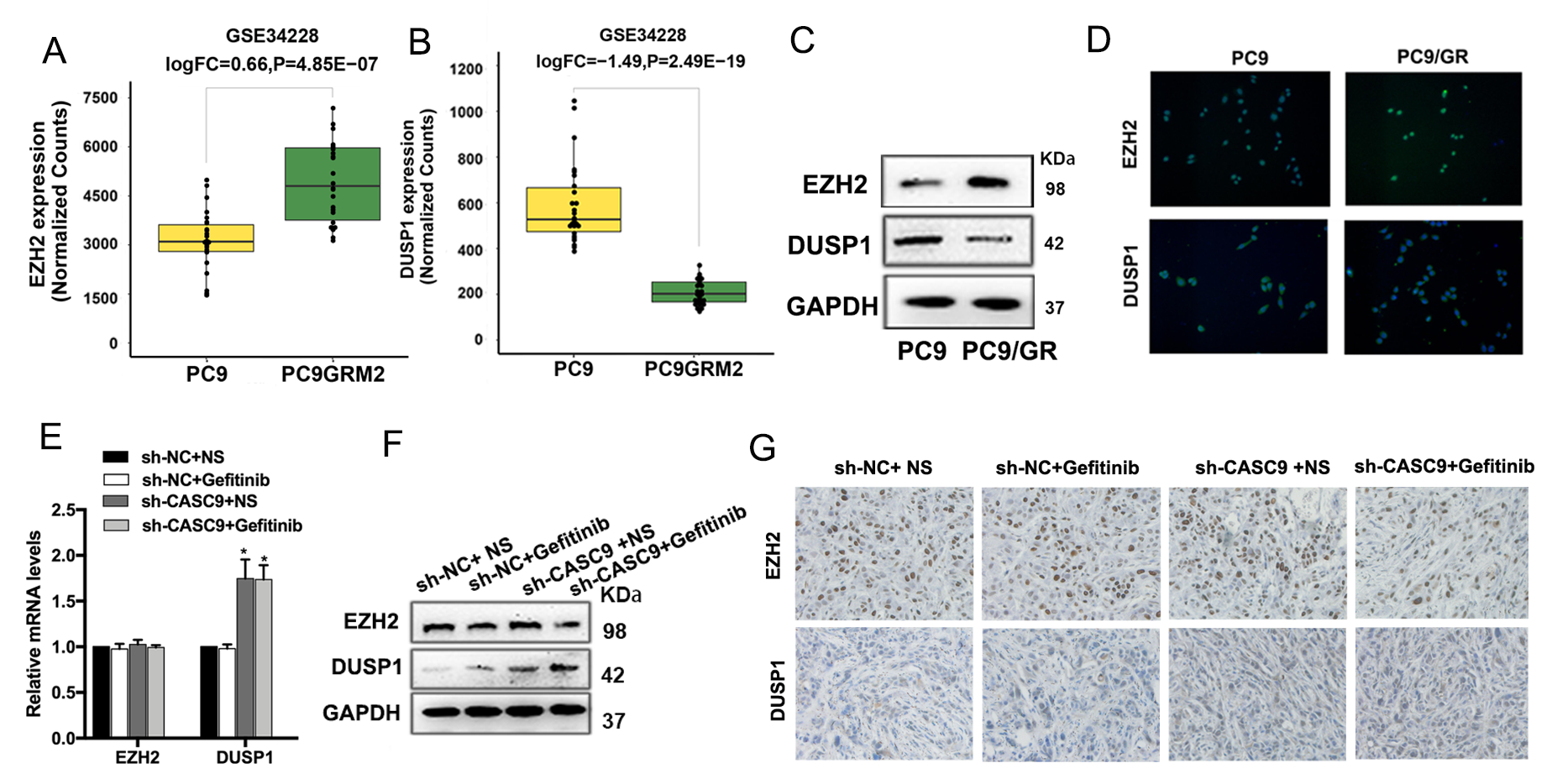

Supplement: Supplementary file 2 — Supplementary Figure S2 [file 41419_2020_3047_MOESM2_ESM.tif]
