# Supplementary material for: Long non-coding RNA CASC9 promotes gefitinib resistance in NSCLC by epigenetic repression of DUSP1
Source: Cell Death Dis. 2020 Oct 14;11(10):858. doi: 10.1038/s41419-020-03047-y (PMC7560854; doi:10.1038/s41419-020-03047-y)
Supplement: Supplementary file 3 — Supplementary Figure legends [file 41419_2020_3047_MOESM3_ESM.docx]

**Supplementary Information**

Supplementary Fig. S1 The role of CASC9 in gefitinib resistant cells.

a. The protein level of p-EGFR, p-AKT, p-ERK, total EGFR, AKT, ERK, and GAPDH in PC9 and PC9/GR cells treated with 5 μM gefitinib were analyzed. b. The inhibition of CASC9 (mRNA) by si-CASC9 was reversed by CASC9 overexpression vector with binding sites of si-CASC9 mutated, based on qRT-PCR. c. The suppression of IC50 value for PC9/GR cells by si-CASC9 was reversed by CASC9 overexpression vector with binding sites of si-CASC9 mutated. d. The antisense oligonucleotide (ASO) specially targeting CASC9 were synthesized to silence the CASC9 expression. e and f. qRT-PCR and western blot assays detected the expression of EZH2 after knockdown of CASC9 in PC9/GR cells. **P* < 0.05, ***P* < 0.01.

Supplementary Fig. S2 Expression of EZH2 and DUSP1 in cells and xenograft tissues.

a and b. EZH2 and DUSP1 expression in gefitinib resistant cells compared with gefitinib sensitive cells analyzed using GEO dataset (GSE34228). c and d. Western blot and immunofluorescence were used to detect the expression of EZH2 and DUSP1 in PC9 and PC9/GR cells. e, f, and g. mRNA and protein levels of EZH2 and DUSP1 were analyzed in the xenograft tumors after sh-CASC9 and gefitinib treatment, based on qRT-PCR, western blot and immunohistochemistry assays.
