# Supplementary material for: Long non-coding RNA CASC9 promotes gefitinib resistance in NSCLC by epigenetic repression of DUSP1
Source: Cell Death Dis. 2020 Oct 14;11(10):858. doi: 10.1038/s41419-020-03047-y (PMC7560854; doi:10.1038/s41419-020-03047-y)
Supplement: Supplementary file 4 — Supplementary Table S1 [file 41419_2020_3047_MOESM4_ESM.docx]

Table1: Clinical characteristics of the 24 patients with EGFR-mutant before ERGR-TKI treatment (BT group) and 18 patients with acquired resistance to ECFR-TKIs (AR group)

| Clinical characteristics | BT group(n=24) | AR group(n=18) |
| --- | --- | --- |
| Sex |  |  |
| Male | 9 | 8 |
| Female | 15 | 10 |
| Age |  |  |
| <60 | 13 | 12 |
| ­­≥60 | 11 | 6 |
| Histology |  |  |
| Adenocarcinoma | 21 | 18 |
| Non-adenocarcinoma | 3 | 0 |
| Smoking |  |  |
| Never | 13 | 11 |
| Ever | 11 | 7 |
| Stage |  |  |
| ⅢB | 5 | 3 |
| Ⅳ | 19 | 15 |
| EGFR |  |  |
| 19DEL | 16 | 5 |
| L858R | 8 | 4 |
| T790M | 0 | 9 |
